# Ancillary Benefit of Microbiology Culture Diagnostic Stewardship: Decreasing Health Care's Climate Impact

**DOI:** 10.1093/ofid/ofae368

**Published:** 2024-07-03

**Authors:** Raja J Hofmeister, Mark D Gonzalez, Jonathan Hildreth, Troy Savage, Amy Leber, Preeti Jaggi

**Affiliations:** The Paideia School, Atlanta, Georgia, USA; Children’s Healthcare of Atlanta, Atlanta, Georgia, USA; Children’s Healthcare of Atlanta, Atlanta, Georgia, USA; Mazzetti Corporation, Atlanta, Georgia, USA; Department of Pediatrics, Department of Pathology and Laboratory Medicine, Nationwide Children’s Hospital, Columbus, Ohio, USA; Children’s Healthcare of Atlanta, Atlanta, Georgia, USA; Department of Pediatrics and Children's Healthcare of Atlanta, Emory University, Atlanta, Georgia, USA

**Keywords:** diagnostic stewardship, greenhouse gas emissions, microbiology cultures

## Abstract

We calculated weights of blood, urine, and endotracheal tube culture supplies and quantified greenhouse gas emissions emitted for disposal of these supplies. Of note, disposal-related emissions for positive blood and endotracheal tube culture results are equivalent to driving 0.6 and 0.4 miles, respectively, in a gasoline-powered vehicle.

The US health care system accounts for 8.5% of US greenhouse gas emissions (GHGe); unnecessary testing contributes to the GHGe attributable to health care [1]. Diagnostic stewardship has been recognized as an important way to prevent unnecessary medications and additional testing and to limit the costs of medical care. The most common type of inappropriate microbiological testing is overtesting [2]. While there are multiple approaches to decrease unnecessary emissions in health care [3], microbiology laboratories dispose of a large amount of waste for every routine culture. Disposal begins with autoclaving and energy-intensive transport to the landfill, and then there are methane emissions from the landfill. We sought to audit and weigh the supplies associated with routine blood, urine, and endotracheal tube (ETT) cultures and to quantify the GHGe associated with the disposal of these cultures. In addition, we estimated the GHGe savings per calendar year from prior published diagnostic stewardship efforts.

## METHODS

This study did not include factors necessitating patient consent and is considered nonhuman subjects research.

### Estimation of Weights of Microbiology Supplies for Routine Cultures

We audited and weighed routine materials used for blood, ETT, and urine cultures at the microbiology laboratories at Children's Healthcare of Atlanta and Nationwide Children's Hospital. “Waste” was defined as anything disposable that was needed to perform the culture, including glass slides for gram stains, agar plates, an assumed volume of specimen, supplies required for species-level identification, and antimicrobial susceptibility testing (Table 1). For the purposes of this project, we considered a positive culture result one that requires further supplies for bacterial identification and antimicrobial susceptibility testing, whether this was clinically required or not. For blood cultures, we included rapid molecular blood culture identification materials. For Children’s Healthcare of Atlanta, either water or the BBL Prompt System (Becton Dickinson) is used for antimicrobial susceptibility testing, so we used the average weight of these 2 items for the weight estimation. We applied conservative estimates of supplies needed by assuming (1) that only 1 of 2 blood culture bottles grew; (2) that there were no mixed flora specimens, so no subcultures were needed for all types of positive culture results; and (3) that urine culture was from a clean-catch specimen, not a catheterized specimen.

**Table 1.** Weights of Microbiology Culture Supplies and Greenhouse Gas Emissions Generated From Disposal

	Urine		Blood		ETT	
Supplies: Laboratory	–	+	–	+	–	+
Gram stain slide,^a^ g						
Laboratory 1^b^	…	…	…	9.4	9.4	9.4
Laboratory 2^c^	…	…	…	4.7	4.7	4.7
Agar plates, g						
Laboratory 1^d^	56.8	85.2	…	113.6	113.6	142
Laboratory 2 ^b,d^	31.2	55.4	…	88	88	117.3
Blood culture bottles, g						
Laboratory 1^e^	…	…	125.4	125.4	…	…
Laboratory 2^f^	…	…	131.2	131.2	…	…
Vent to enter bottle, g						
Laboratory 1^g^	…	…	…	3.98	…	…
Laboratory 2^f^	…	…	…	4.6	…	…
Urine collection vessel, g						
Laboratory 1^d^	16.2	16.2	…	…	…	…
Laboratory 2^h^	17	17	…	…	…	…
ETT collection vessel, g						
Laboratory 1^d^	…	…	…	…	35.4	35.4
Laboratory 2^d^	…	…	…	…	35.4	35.4
Body fluids, 1 mL = 1 g						
Laboratory 1	10	10	10	10	2	2
Laboratory 2	10	10	10	10	2	2
Disposable loop						
Laboratory 1, No.; weight, g^d^	0.92	0.92	0.92	0.92	0.92	0.92
Laboratory 2, 10 or 100 μL, g^c^	1.9	2.83	…	0.8	1.9	1.9
Tube for injection						
Laboratory 1	…	…	…	…	…	…
Laboratory 2	…	…	…	10.9	…	…
Tuberculin syringe to enter bottle						
Laboratory 1	…	…	…	…	…	…
Laboratory 2	…	…	…	3.9	…	…
Pipette, g						
Laboratory 1^i^	…	…	…	…	2.5	2.5
Laboratory 2^c^	…	…	…	…	2	2
Molecular identification kit, g						
Laboratory 1^j^	…	…	…	30	…	…
Laboratory 2^j^	…	…	…	30	…	…
Syringe/tube						
Laboratory 1: SafetyGlide syringe, g^g^	…	…	…	4.78	…	…
Laboratory 2: Tube for injection	…	…	…	10.8	…	…
Alcohol prep pad, g						
Laboratory 1^c^	…	…	…	0.72	…	…
Laboratory 2^c^	…	…	…	0.72	…	…
Vitek 2 AST card and packaging						
Laboratory 1	…	…	…	…	…	…
Laboratory 2^f^	…	21	…	21	…	21
Tube/cap with fluid						
Laboratory 1	…	…	…	…	…	…
Laboratory 2^c,f^	…	5.7	…	5.7	…	5.7
MicroScan supplies,^k^ g						
Laboratory 1	…	90	…	90	…	90
Laboratory 2	…	…	…	…	…	…
Inoculator transfer,^k^ g						
Laboratory 1	…	26.4	…	26.4	…	26.4
Laboratory 2	…	…	…	…	…	…
Media, g						
Laboratory 1	…	50.9	…	50.9	…	50.9
Laboratory 2	…	…	…	…	…	…
Total weight per culture, g						
Laboratory 1	83.92	279.62	136.32	466.1	163.82	359.52
Laboratory 2	60.1	111.93	141.2	322.32	134	190
Greenhouse gas emissions, kg CO_2_ equivalent						
Laboratory 1	0.05	0.16	0.08	0.26	0.09	0.2
Laboratory 2	0.03	0.06	0.08	0.18	0.08	0.11
Miles driven equivalent in gas-powered vehicle per culture						
Laboratory 1	0.12	0.40	0.20	0.67	0.24	0.52
Laboratory 2	0.09	0.16	0.20	0.46	0.19	0.27

Laboratory 1, Children's Healthcare of Atlanta; laboratory 2, Nationwide Children's Hospital (NCH). For urine at NCH, a biplate agar was used (31.25 g/plate), but for blood and endotracheal tube cultures, a single agar was used (29.3 g/plate). Cells without values indicate that the material was not used or needed for this type of culture.

Abbreviation: ETT, endotracheal tube.

^a^Laboratory 1 uses slides in duplicate, and laboratory 2 uses single slides.

^b^Remel.

^c^Fisher Scientific.

^d^Cardinal Health and 28.4 g per agar plate.

^e^BACTEC (Plus Aerobic/F and Lytic/10 Anaerobic/F plastic bottles; Becton, Dickinson and Company).

^f^bioMérieux.

^g^Becton, Dickinson and Company.

^h^Medline.

^i^Copan Diagnostics.

^j^BioFire Diagnostics. Aluminum canister was recycled in both locations and thus not included in the final weight.

^k^Includes additional disposable MicroScan supplies (Beckman Coulter).

### Estimation of GHGe Saved by Intervention

We applied the average weights of the waste in the 2 laboratories as calculated in Table 2 to the following published diagnostic stewardship studies to calculate the GHGe saved with each intervention.

**Table 2.** Average Weights of Microbiology Culture Supplies and Greenhouse Gas Emissions Generated From Disposal

	Urine		Blood		ETT	
	–	+	–	+	–	+
Total weight, g	72.01	195.775	138.76	394.21	148.91	274.76
Greenhouse gas emissions, kg CO_2_ equivalent	0.04	0.11	0.08	0.22	0.08	0.15
Miles driven equivalent in gas-powered vehicle per culture	0.10	0.28	0.20	0.57	0.21	0.39

Abbreviation: ETT, endotracheal tube.

#### Study A: Bright STAR Collaborative for Blood Culture Stewardship

In this 14-center pediatric intensive care unit study [4], blood cultures decreased from 1564 per month to 1130 per month, a savings of 5208 blood cultures per calendar year. We assumed that 2% of blood culture results would have been positive and 98% negative based on prior estimates [5], totaling 104 positive and 5104 negative culture results.

#### Study B: Diagnostic Stewardship of ETT Cultures in a Pediatric Intensive Care Unit

In this single-center study [6], ETT cultures were reduced from 46 to 19 per month, for an average decrease of 27 ETT cultures per month. We assumed that 95% would have been negative and 5% positive, for a total of 16 positive and 308 negative culture results in a calendar year.

#### Study C: Health System Reflex Urine Culture Implementation

Reflex urine cultures were instituted for urinalyses with >10 white blood cells per high-power field, saving 1500 cultures in the 1-year postimplementation period [7]. We estimated that 2% of cultures would have been positive from prior reports of asymptomatic bacteriuria, which would be 30 positive and 1470 negative. Urine collection cup weight was excluded for this study, as this would not have saved the need for the urine collection vessel.

#### Study D: Single-Center Decrease in Overall Urine Cultures Sent in an Intensive Care Unit

The number of urine cultures decreased from 3081 in 2015 to 1218 in 2017, a savings of 1863 cultures per year [8], which would be 37 positive and 1826 negative, assuming 2% positivity as previously described.

### Estimation of GHGe Saved by Weight

Full assumptions and calculations of GHGe from transport, autoclaving, and the landfill are presented in Supplementary Table 1. The Environmental Protection Agency calculator [9] was used to calculate the equivalent miles driven and gallons of gasoline used for context.

## RESULTS

Estimated weights of supplies for routine cultures in the microbiology laboratories are summarized in Table 1. Average weights from the 2 laboratories are presented in Table 2. As expected, a positive blood culture result required more weight than a negative culture (394.2 g vs 138.8 g.). A positive ETT culture result required 274.8 g as compared with 148.9 g for a completely negative culture.

We applied the weights and GHGe derived at our center to the savings from the 4 previously published diagnostic stewardship studies. Estimated savings of GHGe by weight were as follows:

Study A (single-unit, multicenter study) [4]: 749.2 kg of solid waste (419.6 kg CO_2_ equivalent), which translates to a greenhouse-gas equivalent of 1073 miles driven by the average gasoline-powered passenger vehicle, or 47.2 gallons of gasoline consumed.

Study B (single-unit, single-center study) [6]: 50.3 kg of solid waste (28.1 kg CO_2_ equivalent), which translates to a greenhouse-gas equivalent of 71.9 miles driven by the average gasoline-powered passenger vehicle, or 3.2 gallons of gasoline consumed.

Study C (system-wide study) [7]: 86.8 kg of solid waste (48.6 kg CO_2_ equivalent), which translates to a greenhouse-gas equivalent of 124 miles driven by the average gasoline-powered passenger vehicle, or 5.5 gallons of gasoline consumed.

Study D (single-unit, single-center study) [8]: 138.7 kg of solid waste (77.7 kg CO_2_ equivalent), which translates to a greenhouse gas equivalent of 199 miles driven by the average gasoline-powered passenger vehicle, or 8.7 gallons of gasoline consumed.

## DISCUSSION

Each year, millions of microbiological cultures are sent, which results in a considerable amount of waste. Infectious diseases practitioners' efforts have prevented unnecessary cultures from being sent with diagnostic stewardship, which provides clinical benefits to patients. Less recognized are the environmental benefits of these efforts. We calculated the energy burden that was required for just the disposal of routine cultures: positive blood culture results required the equivalent of driving an average of 0.6 miles per culture and positive ETT culture results, an average of 0.4 miles per culture. For 1 patient in the intensive care unit who is “pan-cultured,” this is the equivalent of driving 0.5 miles, assuming that urine, blood, and ETT culture results are all negative. Of note, this was not a complete life cycle analysis of emissions needed to produce these supplies but rather included only the analysis of the GHGe required to dispose of microbiology waste. We also did not quantify the other pollutants associated with disposal (eg, particulate matter); these results should be considered conservative estimates of environmental savings by eliminating unnecessary cultures, as the estimation of the supplies was only for disposal and not the environmental costs of producing the materials needed for culture. Regulated medical waste is generally incinerated or autoclaved in the United States, and we assumed autoclaving. The largest portion of the emissions, however, was associated with methane production from the landfill.

In the United Kingdom, a “sustainable quality improvement” framework [10] has emerged that aims for the best patient outcomes as well as the least social, environmental, and financial impacts of care. Clinicians in the United Kingdom have successfully documented the environmental benefits of their work. In this study, we aimed to empower those involved in creating diagnostic stewardship initiatives with data to inform them of the impact that diagnostic stewardship activities have on GHGe.

A consensus panel recently supported the practice of reflex urine cultures being performed only for those with an abnormal urinalysis finding; thus, this practice could be considered a metric by which hospital systems could be evaluated from a clinical and sustainability perspective [11]. Absence of leukocyte esterase and pyuria has a high negative predictive value for clinically significant bacteriuria [12], and several centers have already implemented urinalysis with reflex urine cultures. As demonstrated in our analysis, this can result in GHGe savings, which are continued yearly, and can save significant amounts of technician time and resources.

Limitations of this study were primarily based on assumptions required to calculate GHGe. We provided these assumptions for researchers who would like to replicate these data so that, if needed, they could modify data to input according to local information and future energy sourcing. The culture positivity rate was based on estimations from microbiology personnel (M. D. G. and J. H.). Strengths of the study include that it reflects 2 susceptibility testing platforms and blood culture system platforms. We did not include other supplies, such as gloves and collection materials, and we assumed that only 1 of 2 blood culture bottles grew an organism. These waste assumptions are conservative, and because this analysis does not include production-associated emissions, these findings could also be considered conservative estimates of the environmental costs of these cultures. There was some variation between the 2 laboratories in the amount of waste generated during the susceptibility testing, but weights of waste generated for negative culture results were relatively similar. The potential benefit of diagnostic stewardship is most likely to save the waste from negative culture results, as unnecessary cultures would have a high pretest likelihood of being negative.

In conclusion, we estimated the GHGe savings from diagnostic stewardship initiatives of blood, urine, and ETT cultures. Infectious diseases clinicians, antibiotic stewards, and infection preventionists may consider using these data to estimate the environmental impact of their work.

## Supplementary Material

ofae368_Supplementary_Data

## Supplementary Data

Supplementary materials are available at *Open Forum Infectious Diseases* online. Consisting of data provided by the authors to benefit the reader, the posted materials are not copyedited and are the sole responsibility of the authors, so questions or comments should be addressed to the corresponding author.

## Note

***Potential conflicts of interest***. All authors: No reported conflicts.
