# Supplementary material for: Ancillary Benefit of Microbiology Culture Diagnostic Stewardship: Decreasing Health Care's Climate Impact
Source: Open Forum Infect Dis. 2024 Jul 3;12(2):ofae368. doi: 10.1093/ofid/ofae368 (PMC11825991; doi:10.1093/ofid/ofae368)
Supplement: ofae368_Supplementary_Data [file ofae368_supplementary_data.docx]

| Source of Greenhouse Gas Emissions | Description and Justification | Assumptions |
| --- | --- | --- |
| CO_2_ Emissions From Transportation | Distance From Hospital to Autoclaving Location (roundtrip in miles)^a^ | 60 miles |
|  | Distance From Autoclaving Location to Landfill (roundtrip in miles)^a^ | 60 miles |
|  | Truck miles per gallon^b^ | 7 |
|  | Fuel Type Emission Factor (kg CO_2_/gallon)^c^ | 8.78 |
|  | Truck Capacity (kilograms)^d^ | 13607.8 |
|  | Attributable Emissions % | 0.0073487% |
|  | *Total Transport Emissions (kg CO_2_)* | *0.0111* |
| CO_2_ Emissions From Autoclaving Process | Autoclaving Electricity Usage (kwh/ton)^e^ | 44 |
|  | Autoclaving Electricity Usage (MWh/ton) | 0.044 |
|  | Electricity Emission Factor (lbs/MWh)^f^ | 891.9 |
|  | *Total Autoclaving Emissions (kg CO_2_)^c^* | *0.02* |
| CO_2_ Equivalent Emissions From Landfill | *Total Landfill Emissions (kg CO_2_Equivalent)* | *0.53* |
|  | **Total (kg CO_2e_)** | **0.56** |

**Supplemental Table 1. Greenhouse Gas Emissions Calculation for Disposal of 1 kg of Microbiology Waste**

**a-Distances are estimated for contracted services from one Children’s Healthcare of Atlanta facility to local disposal sites**

**b-**[**https://www.tandfonline.com/doi/full/10.1080/10962247.2014.990587**](https://www.tandfonline.com/doi/full/10.1080/10962247.2014.990587)

**c-** [**https://www.epa.gov/system/files/documents/2023-03/ghg-emission-factors-hub.xlsx**](https://www.epa.gov/system/files/documents/2023-03/ghg-emission-factors-hub.xlsx)

**d-Cargo estimates from** [**https://www.Bigtruckrental.com**](https://www.bigtruckrental.com/)

**e-Assumption used in Mazzetti M+Wastecare** [**https://wastecare.mazzetti.com/calculator**](https://wastecare.mazzetti.com/calculator)

**f-Based on zip code 30322 electric grid subregion** [**https://www.epa.gov/egrid/power-profiler#/**](https://www.epa.gov/egrid/power-profiler#/)
